# The impact of hyperhidrosis on patients' daily life and quality of life: a qualitative investigation

**DOI:** 10.1186/s12955-017-0693-x

**Published:** 2017-06-08

**Authors:** P. Kamudoni, B. Mueller, J. Halford, A. Schouveller, B. Stacey, M.S. Salek

**Affiliations:** 1 Institute of Medicines Development, Duffryn House, Cardiff, CF 23 6NP UK; 2 Medical Science and Operations Department, Riemser Pharma GmbH, Greifswald, Germany; 3 The Hyperhidrosis Support Group UK, http://www.hyperhidrosisuk.org; 4 Hyperhidrosis Patient Forum, http://www.verysweatybetty.com; 5 Department of pharmacy, School of Life and Medical Sciences, University of Hertfordshire, College Lane, Hatfield, Herts UK

**Keywords:** Hyperhidrosis, Excessive sweating, Daily life impacts, Quality of life

## Abstract

**Background:**

An understanding of the daily life impacts of hyperhidrosis and how patients deal with them, based on qualitative research, is lacking. This study investigated the impact of hyperhidrosis on the daily life of patients using a mix of qualitative research methods.

**Methods:**

Participants were recruited through hyperhidrosis patient support groups such as the Hyperhidrosis Support Group UK. Data were collected using focus groups, interviews and online surveys. A grounded theory approach was used in the analysis of data transcripts. Data were collected from 71 participants, out of an initial 100 individuals recruited.

**Results:**

Seventeen major themes capturing the impacts of hyperhidrosis were identified; these covered all areas of life including daily life, psychological well-being, social life, professional /school life, dealing with hyperhidrosis, unmet health care needs and physical impact.

**Conclusions:**

Psychosocial impacts are central to the overall impacts of hyperhidrosis, cutting across and underlying the limitations experienced in other areas of life.

**Electronic supplementary material:**

The online version of this article (doi:10.1186/s12955-017-0693-x) contains supplementary material, which is available to authorized users.

## Background

Primary hyperhidrosis is characterized by spontaneous excessive sweating beyond the physiological needs of the body [1], with a prevalence of 2.8% in the United States of America (USA) [2], 9.3% in Germany (for focal hyperhidrosis) [3], and 2.79-5.75% in Japan [4]. According to previous studies in Germany, Brazil and Japan, 30-37.9% of patients with hyperhidrosis are frequently or constantly bothered by their sweating, resulting in impairment in daily activities, mental health and study or work life [3–5].

Patients with hyperhidrosis experience impairment in various functional domains as well as in overall quality of life. Several studies in hyperhidrosis patients awaiting surgery reported reduced vitality and mental health according to the short-form survey 36 (SF-36) questionnaire [6–8]. Various impairments were reported in a study employing the Illness Intrusiveness Rating Scale (IIRS) questionnaire including, work limitations, disturbances in social life, relationship with spouse and recreational activities [9]. Other studies [1, 2, 10] have reported reduced effectiveness at work (34-79% of patients) and moderate to severe emotional impact (64.7- 86%) such as depression, feeling unhappy, reduced confidence. Studies [1, 11–23] employing the dermatology life quality index (DLQI) suggest a moderate to very large effect on the patient’s life [24]; scores reported ranged from 10 to 14 for axillary hyperhidrosis, 8.8–15 for palmar hyperhidrosis, 13 for craniofacial hyperhidrosis, 9.4 for the trunk across 14 studies in hyperhidrosis patients.

The only previous study employing qualitative methods (i.e. focus groups) and including female patients only [25] reported various impacts including: low self-esteem, psychological distress, worries about clothes getting stained, a loss of control over own life as a result of hyperhidrosis.

A comprehensive understanding of the extent and nature of the daily life and quality of life impacts of hyperhidrosis across all forms of the condition based on qualitative methods is still lacking. This would offer key insights into factors confounding patient outcomes, the long-term consequences of impairment and how patients deal with the condition. Moreover, quality of life issues specific to non-clinic hyperhidrosis patients are sparsely understood. The aim of this study, therefore, was to investigate the impact of hyperhidrosis on the quality of life of individuals with hyperhidrosis using a mix of qualitative methods including interviews, focus groups and online surveys for data collection.

## Methods

Study participants included members of hyperhidrosis patient support groups including the UK Hyperhidrosis Patient Support group*,* the Very Sweaty Betty and other groups. Inclusion criteria included: age of 16 years or greater; self-reported hyperhidrosis; seeking for treatment; and ability to communicate in English. Exclusion criteria included excessive sweating related to a specific health issue (based self-report or late onset of sweating combined with the presence of comorbidities or use of medication known to be associated with sweating).

Ethics approval for this study was obtained from the ethics committee of the University Hospital of Greifswald, Germany. Informed consent was obtained from all participants electronically or via wet-ink copy (sent by post or Email) before their participation in the study.

### Procedure

Purposive sampling and snowball sampling were employed in the current study. The sampling strategy aimed at including patients with hyperhidrosis of all severity levels as well as all areas of involvement, and covering different demographic characteristics. Participants were asked to share information about the study in their social media networks (if they were willing to do so), making the most of the typical usage of social media (e.g. Facebook) for recruiting more participants. During the recruitment phase, the research team collaborated with administrators of online social networks to recruit their members; these were either patients with hyperhidrosis themselves or volunteers supporting the cause of hyperhidrosis. Thus, the participant recruitment process was led by such patient partners. The appropriate sample size for the study could not be determined at the outset but was rather determined during data collection, based on reaching conceptual saturation i.e. a point at which further data collection does not yield new concepts or information [26].

Information about the study was posted on the study’s Facebook page and on other online patient communities for hyperhidrosis. Those contacting the research team and fulfilling the screening criteria were sent a patient information sheet and were recruited into the study. Data collection took place between April and August 2011, and employed qualitative methods including focus group discussions, semi-structured interviews and online surveys (with open-ended questions). Qualitative methods are particularly useful for obtaining insights into subjects’ beliefs, views and understanding of particular phenomena [27].

The choice of participant recruitment and data collection approaches in the study was informed by key features of hyperhidrosis such as low prevalence rate and a small proportion of patients seeking professional help, among other considerations. Use of online social networks for reaching patients addressed some of the hurdles of doing research in this population.

Integration of multiple methods of data collection, also known as triangulation, was employed to enhance the validity of the collected data [28]. For example, data generated from the focus groups were further explored in depth during the interviews. Subsequently, the key themes from the focus groups and interviews were confirmed in the surveys. The three methods have different strengths. Interviews allow for in-depth insights into participant’s experiences and allow for greater self-disclosure [29]. Focus groups facilitate interaction among participants which produces unique data providing a perspective on consensus or contentious issues [29]. Surveys (with open-ended questions) allow better exploration of heterogeneity in experiences of phenomena through a larger sample. Thus, combining the different methods allowed the study to benefit from the strengths of the different approaches while offsetting known limitations.

The focus group discussions were text-based and were carried out asynchronously via an online discussion board developed specifically for the study. Each focus group was run for up to 2 weeks. A member of the research team (P.K.) moderated the discussions by posting questions, comments and probes based on a topic guide while allowing the discussions to flow naturally among the participants. Thus, in addition to responding to moderators prompts, participants also commented on posts from fellow participants.

The semi-structured interviews were carried out via telephone or using instant messaging e.g. Skype*.* All interviews were carried out by the same member of the research team (P.K.) which enhanced consistency of data collection. Similar to the focus groups, the interviews were conducted following a topic guide, developed based on previous research and input from clinical experts. At the beginning of the interviews, participants were invited to describe their experiences of living with hyperhidrosis, using an open ice-breaker question. Participants were also prompted on issues previously reported to be important to hyperhidrosis patients, where the participant did not initiate discussion voluntarily.

The online survey was developed based on results from the focus group discussion and interviews and included open-ended questions. A URL link to the surveys was posted on the portals for the UK hyperhidrosis society and other relevant forums.

### Analysis

Interviews were tape-recorded then transcribed verbatim. The focus group discussions and open surveys were already in text format. Applying a modified grounded theory approach [30], data transcripts were analysed to identify key themes and concepts in a data-driven inductive process, with the concepts and structure emerging from the data. Prior knowledge including previous research and expert input had some influence on the data collected, for example in the development of the topic guide, and in the research team’s early understanding of the impacts of hyperhidrosis. The analysis process was iterative, involving a studying the primary data and developing interpretations and concepts, carrying out further data collection, and revising earlier interpretations and developing new concepts.

Data coding was applied during the analysis and was performed by one member of the research team (P.K.) under the supervision of a senior researcher (a member of the research team). Initially, a codebook was developed based on analyses of initial data transcripts, this was then discussed with the research team before all the data were coded. The codebook was a living document and was updated throughout the analyses as necessary.

Different coding strategies were employed during the analysis, starting with **open coding**, where events and issues identified from the data transcripts were assigned initial conceptual labels (codes) to facilitate comparisons of similarities and differences between phenomena [31]. Subsequently, the initial codes were reorganised based on their similarities and difference through **axial coding**. Finally, further categorization and organisation of the axial codes was carried out using **selective coding**, to produce the overall conceptual structure (also facilitating the definition of the overall concept (s)). Processing of data transcripts and coding was carried out using QSR International Nvivo 9 software. Further insights into the data were sought by comparing themes across different hyperhidrosis sites and demographic factors.

## Results

Seventy-one patients took part in the study (*n* = 9 for focus groups; *n* = 32 for semi-structured interviews; and *n* = 30 for online surveys), out of an initial 100 individuals recruited. The mean age of participants (males = 21, female = 50) was 35 years (range 16–67) and the mean duration of hyperhidrosis was 23 years (3 to 60 years) (Table 1). The 20 to 29 years’ age group made up 45% of the sample and was the largest group. Participants reporting generalised hyperhidrosis comprised 28% of the sample and represented the most prevalent area of involvement.

**Table 1:** Demographic characteristics of the study participants

Characteristic	Frequency
Gender (%)	
Male	21 (30%)
Female	50 (70%)
Age (years)	
Mean (SD)	34.9 (13.2)
Median	31
Range	16–67
Duration of disease (years)	
Mean (SD)	23.3 (13.6)
Median	21
Range	3–60
Body site affected (n)^a^	
Armpits only	4
Armpits plus other sites	9
Palms only	3
Feet only	2
Palms and feet	14
Plantar, palmar and axillary	14
Generalised	20
Cranium-face	4
Trunk and lower body	1
Country	
U.K.	41
U.S.A	14
Canada	2
Netherlands	2
Other	12

^a^ Each participant is counted in one classification only

Theme saturation occurred on the 33rd patients i.e. no new themes emerged after this point. In consideration of the novelty of the patient recruitment approach employed, further data were collected through interviews and online surveys after this point.

**Fig. 1:**
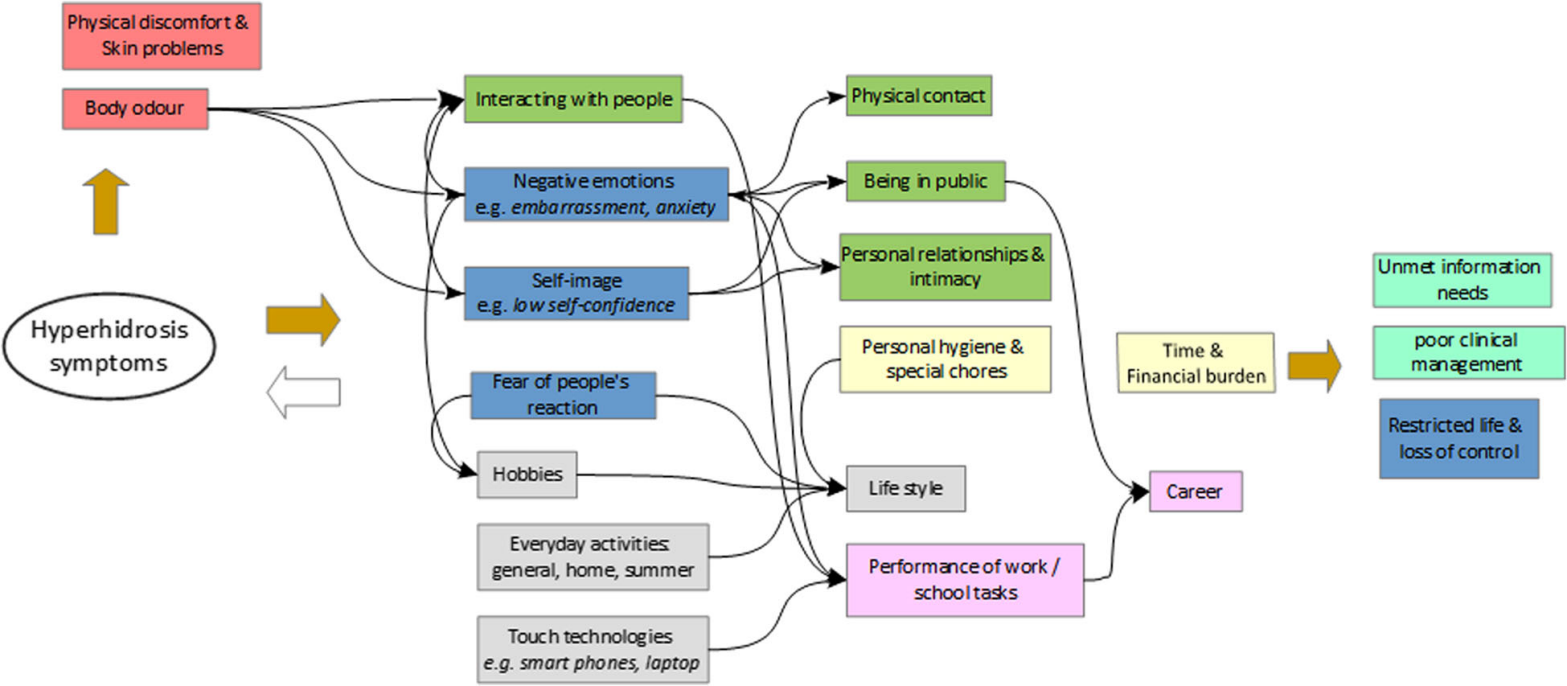
Conceptual framework showing the impacts of hyperhidrosis across different areas of life, and their inter-relationships based on data collected

Seventeen themes covering seven major areas including daily life (reported by 95.8% of participants), psychological life (91.5%), social life (90.1%), professional life (74.6%), dealing with the condition (74.6%), unmet health care needs (64.8%) and physical impact (53.5%) were identified from the data. A conceptual framework showing themes identified in the data, and their inter-relationships is presented in Fig. 1. In addition to the quotes in the main text, additional quotations from the participants are provided as Additional file 1.

### Daily life

#### Lifestyle

Nearly three-quarters of the study participants reported an impact on lifestyle. The majority of patients (61%) mentioned that sweating influenced their choice of clothing, particularly, material, colour and design of clothes. Most participants avoided bright colours such as red or blue, in favour of blacks or whites; and materials such as polyester were avoided in favour of cotton. Participants with plantar hyperhidrosis mostly avoided flip-flops or sandals. Some participants (13%) reported avoiding spicy or hot foods, and drinks containing alcohol or caffeine, while others could no longer enjoy holiday activities such as basking in the sun and were unable to go to warm holiday destinations.

#### Hobbies

Various types of hobbies were affected as a result of the hyperhidrosis, among 41% of the participants. These included physical as well as non-physical activities such as cycling, exercising in the gym, playing musical instruments, doing yoga, and crocheting/knitting. One patient said, “*I don’t like exercising on the street or anything like that, […] going for a walk, […] people tend to look at me if am really sweaty and that makes me really nervous*”. For example, reading books or newspapers had become difficult, because of the challenge of holding books without getting the pages soaked, especially for patients with palmar hyperhidrosis. Effects on hobbies were a direct result of the sweating as well as a secondary consequence of impacts in other areas e.g. avoidance of embarrasing situations.

#### Everyday activities

Hyperhidrosis interfered with general daily life activities such as household chores, and summer time activities for most patients. Activities of greatest concern to the participants were difficulties with holding objects, turning door knobs, opening jars, working with hand tools and driving, shopping, and manual work. Impacts related to these activities seemed to be primarily associated with wet palms. For example, one patient said, “*It even affects how I pay for things. I don't use cash anymore unless I know I have the exact change because I don't like getting coins back since the other person usually touches your hand when they give you change back*”. Several patients described their difficulties or discomfort with light activities such as dressing due to sweating.

A third of all participants had problems with daily household chores such as cleaning, cooking, ironing and caring for young children. For example, one participant said, “*the minute I start to do anything the least strenuous I stream with sweat, so housework is a nightmare. I have to change all my soaked clothes if I hoover [vacuum cleaning] one room. I have to tie strips of towel around my forehead and neck when I do anything that involves movement, and have to keep changing them as they get soaked’.* The majority of the participants (73%) reported difficulties with various activities associated with the summer months (in Europe and North America) such as gardening and mowing; doing barbeque; and going to open air concerts. The onset of summer was a cause of concern to some participants, for example, one subject said: “*I have a son who I’d love to take out in summer, but sometimes we don’t because I have so many years of fearing summer*”.

#### Touch technologies

A fifth of the respondents reported problems with using touch technologies such as computer-keyboards, computer-mouse, mobile phones and touch screens interfaces. One patient said, “*I did have to get a special portable mouse ordered that I could plug into my laptop to navigate the programs on the screen, as the built in mousepad does not pick-up touch of my wet fingers. I am hesitant to purchase an Android, Blackberry Touch, or iPhone because of my sweating. If the laptop mouse can’t detect my fingers, how will the phones? I do own an iPod, but occasionally I can’t navigate the scrolling menu if my hands are too we*t.

Across all hyperhidrosis sites, impairments /related to daily life activities often were a secondary consequence of the hyperhidrosis; i.e. resulting from avoidance of activities that would result in sweating, fear of people’s reaction or fear of embarrassment. Generalised, lower trunk, cranial facial hyperhidrosis was not linked to impairment in specific daily life activities, apart from besides the general inconvenience of having to do activities such as housework while excessively sweating. Inconvenience experienced when patients with palmar hyperhidrosis additionally, experienced a direct interference with activities involving the hands.

### Psychological well-being

#### Negative emotions

A majority of the participants (69%) had experienced some emotional sequelae as a result of the hyperhidrosis, and these were the most prevalent psychological effect of hyperhidrosis. Patients were often embarrassed and were often anxious about sweat breaking out at any moment. Other less frequent emotions included sadness, anger, and hopelessness.

Although negative emotions were considered a consequence of hyperhidrosis, their role in exacerbating the severity of sweating and other impacts was also mentioned and highlighted by the participants. For example, one patient said: “*I’m on an anti-depressant, I feel I sweat because I’m nervous and I’m nervous because I sweat. It’s a vicious circle. I feel that anti-depressant helps to relieve that nervousness and helps to relieve a tiny percentage of my sweating*.”

#### Fear of people’s reaction

Concerns related to other people’s negative reaction and judgement were reported by 64% of the study participants. There was a belief that other people’s negative reaction was due to a general lack of understanding about hyperhidrosis among the public. Because of this, most participants reported being constantly worried about how noticeable their sweating was. Further, participants feared leaving behind sweat marks on chairs, doorknobs and other objects. One participant said, “*Last year I plucked up the courage to go shopping for a pair of sandals. As I bent down to put some on, my face and hair dripped so badly onto the floor it created a puddle. I also poured sweat onto the one shoe I’d tried – before leaving the shop and never going back. I was glared at by the manager and assistant*.

#### Self-image

Hyperhidrosis was reported to have extensive consequences on participants' self-image. Most individuals reported having a low self-esteem and self-confidence as a result of hyperhidrosis, while others described how they had become more self-conscious. For example, one participant said, “*I am disgusted in myself for it and so it massively eats away at my self-confidence, it makes me feel awful and dirty and gives me low self-esteem - this has certainly been the root cause of my severe lack of self-confidence in everything I do or I am*”. A small number of participants (15%) were concerned with their appearance and reported feeling less attractive. This was particularly emphasised by female participants. For example, one participant said, “*hyperhidrosis has made me feel less feminine at times. Of course, I know that males with hyperhidrosis also have a hard time coping, but females are expected to sweat even less than males. It has also made me feel less desirable sexually*... *At times in my life, I’ve had low self-esteem*.”

#### Restricted life and loss of control

Half of the sample felt their life had been restricted by hyperhidrosis. One participant said, “*there has been countless things I haven’t done because you first sort of think oh God that’s going to place me in an uncomfortable position*”. Another individual described the experience of living with hyperhidrosis as ‘being trapped’. Some patients talked of hyperhidrosis taking over their life, while others said it was a constant preoccupation. For example, one of the major worries mentioned by a number of patients was about the condition worsening in future.

### Social life

#### Being in social situations

Being in public view was a challenge for three-quarters of the participants. Social situations such as being in a shared office, class-room, going out to a party, restaurant or cinema were challenging. Participants mentioned that they often would end up avoiding such situations altogether. Several individuals described difficulties with travelling on public transport. One participant reported that “*being at work, or travelling to and from work is mortifying. Sitting or standing in sodden clothes for 8 hours and travelling on public transport is horrendous*”. Impacts in other areas such as negative self-image and fear of people’s reactions were often the underlying issue or exacerbating factor in social impacts.

Fifty-seven percent of the study participants had difficulties interacting with others. A heightened self-consciousness, reduced self-confidence and fear of being judged by others made interaction challenging. For example, one participant said, *“[the sweating] affects my social competence because it is difficult focusing on a conversation when trying to hide sweating or thinking about how disgusting it feels against the body and having wet clothes*”. Some individuals had difficulties with meeting new people, due to a lack of confidence and a fear of how others may react to the sweating. One participant reported the following: “*when meeting new people, I have a constant worry and fear of shaking hands. Do I shake their hand? Do I pretend I didn’t see them offering their hand? Do I tell them I have sweaty hands? Endless excuses echo around your head*”.

#### Physical contact

Most participants were uncomfortable with close proximity to others due to fears about their reaction. Hence, they avoided touching, holding or shaking hands, sitting next to others, standing in a queue or dancing. Some participants were uncomfortable with hugging, cuddling or any other forms of physical affection. For example, one patient said, “*you were very aware of it…when people would get close to you…it was really embarrassing…I didn’t really like that…you were afraid that they would notice…that you were sweating*”. Another participant said, “*I am unable to touch my husband, daughters and grandchildren without first thinking about how to do it without them actually having contact with my skin*”.

#### Personal relationships and intimacy

Study participants described negative consequences of hyperhidrosis on their personal relationships. Participants reported that their avoidance of going out and being in public meant that they were not able to nurture important relationships. Others attributed their inability to get new friends or partners to the condition. For example, one participant said, “*I have not been in a relationship, as I feel too embarrassed to explain my excessive sweating, I lost touch with most of my friends after school, because by this time, the sweating had got worse and they were wanting to go out, I would feel too anxious about it and would make an excuse*”.

A few participants described how hyperhidrosis had an impact on their sex lives. One participant said, “*I can’t cuddle my girlfriend quite lovingly...I can’t have sex without thinking about it. To be honest, as soon as I start sweating, I know that am sweating and then I lose my erection because I know that am sweating and I think they know that am sweating*”.

### Dealing with Hyperhidrosis

#### Personal hygiene and special chores

Participants described the burden associated with managing their hyperhidrosis. In addition to taking various treatments, the participants employed various strategies in managing symptoms and impacts of hyperhidrosis. These were described as “little rituals” by one patient, and included carrying around towels, tissues or handkerchief, and a pair of extra clothes for changing; keeping a fan or air-conditioner running when at home or at work; and using hand driers in restrooms when in public buildings.

Seventeen percent of the individuals mentioned doing extra chores in order to stay clean, such as taking a shower several times a day, changing clothes or shoes several times, and using strong deodorants. One participant said, “*I have to wash my uniform each night and sometimes take a spare set to work to change during the day*”.

#### Time and financial burden

Managing the hyperhidrosis was described as time-consuming by the study participants. Individuals were concerned about the time they spent in dealing with hyperhidrosis each day, for treatment, management of symptoms, and for personal hygiene. Performing daily activities such as walking or dressing-up would usually require more time, to avoid a bout of sweat from breaking out. Organising daily life involved planning in advance on things such as clothing and leaving enough time to do things at a slower pace, which meant some loss of spontaneity.

Some participants described concerns over the additional expenditures associated with hyperhidrosis, including out-of-pocket payment for treatments, new clothing and other personal hygiene products /materials.

### Physical impact

#### Physical discomfort and skin problems

Hyperhidrosis was associated with some level of physical discomfort for at least 40% of the participants. In particular, individuals experienced discomfort in relation to ‘*being in wet clothes day in day out’*, ‘*having wet feet’,* and *‘sweat dripping into the eyes’.*

Seventeen percent of the patients reported other skin problems which they attributed to the constant dampness associated with hyperhidrosis. The most common were soreness and cracked skin. For example, some patients considered their hand eczema and athlete’s feet to be caused by the excessive sweating. These were often recurrent reflecting the chronic nature of hyperhidrosis.

#### Body odour

Body odour was an issue for some patients, particularly when in public, in close proximity with others or in enclosed spaces. One participant said, “*The worst is the effect of unpleasant odour of my feet. I remember taking a bus ride and everybody noticed the offending smell. I try to avoid enclosed places like elevators, conference room, and airport lounge*”.

### Unmet healthcare needs

#### Clinical management of condition

Most participants were not satisfied with the way their condition had been managed. In particular, they were concerned about the poor relationship with their doctor, the difficulty of obtaining an accurate diagnosis for the condition, and the lack of or poor access to effective treatments as well as negative side-effects of treatments.

The experience of getting diagnosed was described as “humiliating” and a “belittling experience”. This was especially because the sweating problem was not taken seriously by the clinicians. For example, some participants reported being told that they were wasting their GPs [general practitioner’s] time. For others, diagnosis of the condition had taken long.

Although certain therapies such as Botox and Iontophoresis were considered to be effective, participants were worried about the limited coverage of such treatments. For example, in the UK national health service (NHS) patients would only be allowed a certain number of cycles, and then would have to pay out of pocket thereafter. Those with a history of endoscopic thoracic sympathectomy surgery (ETS) experiencing compensatory sweating were frustrated with the lack of any other effective therapy.

#### Lack of information

Nearly a third of the study participants found currently available information on hyperhidrosis inadequate. During the interviews, participants wanted to find out the cause of their condition and the new treatments under development. Patients also expressed worries about the level of knowledge of health-care practitioners about the condition. Other participants were concerned about the lack of public awareness about the condition and considered this to be the basis for the lack of public sympathy for hyperhidrosis.

Concerns about the lack of effective treatments and access to the available treatments was a generic issue, reported by patients with all hyperhidrosis sites. Patients with hyperhidrosis affecting the lower trunk or generalised over the entire body sweating emphasised the need for information on ETS surgery. Most of these were unaware of the risk of compensatory sweating associated with ETS surgery.

### Professional or school life

#### Work or school tasks

Participants had difficulties with their work because of the hyperhidrosis. Sixty-three percent experienced interference in the performance of tasks at work or school; the majority regarded this as the most important impact of the condition. Having wet palms presented a challenge in performing various tasks, such as writing on paper, using a computer, or operating machinery. For instance, a nurse reported that she found it hard to put on latex gloves or administer an IV to patients. Another patient reported that fear of judgment made dealing with clients and work colleagues a challenge.

#### Career

A third of the participants reported choosing careers that would accommodate their sweating. One participant had let go of an opportunity to become a policeman “settling for a boring office job”. Some participants believed that their condition had hindered their career progression, while a few participants had opted for early retirement because of their condition.

Impacts on work or school life followed a similar mechanism as those on daily activities i.e. these were mostly secondary effects of impacts in other areas. For example, impacts on work productivity were related to participants’ efforts to prevent sweating, or the fear that their sweat would be noticeable and the fear of people’s reaction or embarrassment. Direct interference of sweating with work or school life was mostly particular to palmar hyperhidrosis and was, for example, related to difficulties with using computer keyboards, holding objects, working with paper documents or giving handshakes.

## Discussion

Skin disorders carry far-reaching consequences beyond the clinical signs and symptoms, known and appropriately reported by the patient alone; Chren et al. [32] suggested that such subjective experiences be regarded as part and parcel of vital signs of disease activity. The findings from the current study suggest that the impact of hyperhidrosis on patients' life is broad and affects all areas of life such as daily life, psychological well-being, social life, professional life, dealing with the condition, unmet healthcare needs, and physical impact.

Negative emotions such as anxiety and embarrassment were often highlighted as a reason for avoiding various activities, resulting in handicap in basic daily life activities. For example, shopping and paying for groceries was challenging partly because patients were uncomfortable with being near others or did not want to brush hands with the cashier. Having wet palms and negative emotions was an underlying issue in impacts on professional life. On the extreme, the participants often resorted to avoidance of situations that would aggravate the sweating or the impacts; this may, in turn, have long-term or major consequences, for example, influencing major life-changing-decisions or personalities.

The importance of psychosocial impacts may be further amplified by the multifactorial nature of hyperhidrosis -excessive sweating symptoms are exacerbated by patients psychological response such as negative emotions [33]. It has been shown empirically that anxiety or stress as well as being in social situations are more important aggravating factors for hyperhidrosis than heat or summer season [34]. This reflects the perennial nature of hyperhidrosis.

No major differences were noted in areas of impact such as choice of clothing, fear of leaving sweat marks on objects, impacts on social life, impacts on emotional life, career choices, and hobbies across hyperhidrosis affecting different sites. For example, although patients with hyperhidrosis of different sites used different approaches to deal with symptoms or impact of the condition, the actual burden associated with dealing with the condition appeared to be similar.

A previous study reported that individuals with hyperhidrosis spend 15 to 60 min in managing symptoms of the condition and that 50 to 70% change their clothes more than twice a day [23]. One in every five patients relies on some form of accessory to manage their daily life normally [2]. A good part of dealing with the condition involves disguising or concealing the sweating.

The current findings suggest that individuals with hyperhidrosis have healthcare needs that are currently not being met such as access to treatment, adequacy of patient information, and support in dealing with the psychological scourge of hyperhidrosis. Similar information problems have been noted in other dermatologic conditions such as psoriasis [35]. This suggests that individuals with skin disorders including hyperhidrosis may benefit from interventions helping them deal with the wider impacts of their condition such as counselling, education and psychotherapy, accessible within and outside the clinic. In this context, online platforms may offer a wide scope for increasing the availability of such services. The depth and nature of data collected across the three data collection approaches – focus group discussions, semi-structured interviews and online surveys were different; however, examining such differences was not pertinent to our research objectives. The focus group discussions generated in-depth data and rich descriptions of experiences, although there was a tendency for participants to go off-topic to talk about issues most pertinent to them (e.g. use of different deodorants or armpit pads) but of limited relevance to the research question. On the other hand, the semi-structured interviews were more focused, and resulted in greater self-disclosure. For example, the theme related to sex-life only came up during semi-structured interviews. The online surveys did not generate any new themes, beyond what emerged from the focus group discussions and interviews. The descriptions provided by patients tended to be brief and short, nevertheless, the surveys provided greater heterogeneity in descriptions used for phenomena or concept across participants. Combining the three data collection methods enhanced the validity of the findings from the research, ensuring that the disparate ways a common impact may be experienced as well as unique individual experiences were both captured.

### Strengths

The data collection in the current study benefited from the triangulation of several qualitative data collection methods including focus groups, semi-structured interviews and surveys with open-ended questions. For instance, the interactions among participants during the text-based discussions, the in-depth and more detailed information about the individual experience from interviews, and the possibility of reaching a larger number of patients through online surveys enhanced the reliability and validity of the data collected.

Further, the involvement of patients in a different role besides as research subjects – as research partners, enhanced the patient centredness of this research. In addition to supporting the patient recruitment process, the patient research partners were involved in other steps of the research process, such as the development of the manuscript.

### Limitations

The major limitation of this study was the lack of opportunity of carrying out data collection face to face and in a clinic population. The current patient population was comprised of members of online patient support groups for hyperhidrosis. This, in turn, contributed to the inability of the researchers to authenticate the identity of study participants; thus, our sample may have included patients without the diagnosis of hyperhidrosis, despite a self-reported diagnosis of hyperhidrosis being one of the inclusion criteria. This was partly mitigated by the study participants providing electronic or wet-ink (by post or Email of scanned copy) consent. In addition, all patients provided a narrative about their disease history including how their condition was diagnosed. A disease history consistent with hyperhidrosis provided some validation of their diagnosis.

An internet sample frame means that patients without access to the internet did not have any chance of being included in the current study. While this and the related lack of representativeness may indeed limit the generalisability of the current findings, the current study design enabled the recruitment of a large international sample of hard-to-reach patients (outside clinic settings) which was diverse in terms of site and severity of hyperhidrosis, age and disease duration.

## Conclusions

Our findings show that hyperhidrosis affects all aspects of a patient’s life, with psycho-social effects playing a central role, suggesting that pharmacological therapy alone may not address all the needs of the patient. An integrated approach incorporating various healthcare professionals such as psychiatrists and counsellors would be key in tackling the psychosocial impairment associated with hyperhidrosis. This could be delivered as part of a well-orchestrated patient-centered dermatologic care. Of note, a new patient-reported outcome measure of the quality of life specific for hyperhidrosis, the Hyperhidrosis Quality of Life Index [36], has recently been developed based on the findings from the current study. It is envisaged that the new measure will play a pivotal role in improving the care of hyperhidrosis particularly addressing the information needs as well as making the patient-doctor interaction more patient-centered.

## Additional file

Additional file 1: Quotations from participants. (DOCX 26 kb)

## Abbreviations

DLQI: Dermatology Life Quality Index
ETS: Endoscopic Thoracic Sympathectomy
IIRS: Illness Intrusiveness Rating Scale
NHS: National Health Service
SF-36: 36-item short form health survey
UK: United Kingdom
USA: United States of America
