# Supplementary material for: The impact of hyperhidrosis on patients' daily life and quality of life: a qualitative investigation
Source: Health Qual Life Outcomes. 2017 Jun 8;15:121. doi: 10.1186/s12955-017-0693-x (PMC5465471; doi:10.1186/s12955-017-0693-x)
Supplement: Additional file 1: — Quotations from participants. (DOCX 26 kb) [file 12955_2017_693_MOESM1_ESM.docx]

**THE IMPACT OF HYPERHIDROSIS ON PATIENTS’ DAILY LIFE AND QUALITY OF LIFE: A QUALITATIVE INVESTIGATION**

**SUPPLEMENTARY MATERIALS: QUOTATIONS FROM THE PARTICIPANTS**

P. Kamudoni^1^, B. Mueller.^2^, J. Halford^3^, A. Schouveller ^3^, B. Stacey ^4^, M.S. Salek^1,5^

1. Institute of Medicines Development, Cyncoed, Cardiff, UK.

2. Formerly, Medical Science and Operations Department, Riemser Pharma GmbH, Greifswald, Germany (formerly).

3. The Hyperhidrosis Support Group UK, [www.hyperhidrosisuk.org](http://www.hyperhidrosisuk.org)

4. Hyperhidrosis Patient Forum, [www.verysweatybetty.com](http://www.verysweatybetty.com)

5. Department of pharmacy, School of Life and Medical Sciences, University of Hertfordshire, College Lane, Hatfield, Herts, UK.

Dr Paul Kamudoni

Institute of Medicines Development,

Duffyrn House,

Cardiff,

CF 23 6NP,

UK.

Tel : 07768529783

Email : [pkamudoni@gmail.com](mailto:pkamudoni@gmail.com)

**QUOTATIONS FROM PARTICIPANTS**

**Daily life**

Lifestyle:

“It may sound silly...but I never ever wear sandals...I wear closed shoes in the summer...I wear mainly cotton footsore...they are God send...it’s not only the soles of my feet that sweat it’s the top of my toes...as well...so if I would put sandals on and people see the glittering that makes me worse it’s just more of an embarrassing thing.” (No.7, Female, 57 years, palmar-plantar).

Everyday activities:

“activities that are very uncomfortable for me are playing certain games with my kids, shopping for clothes because I come out of the dressing room drenched in sweat. Holding my husband and kids hand” (No.36, Female, 33 years, palmar-plantar).

“I once cut my fingers after my hand slipped trying to get a vacuum attachment hooked from the vacuum, difficult to wear rubber gloves when cleaning since they get all sweaty” (No.26, Female, 32 years, palmar-plantar).

Touch technologies

“Can’t use a mousepad on a laptop, must have a wireless mouse since the pad can’t detect my fingers; keyboard get sweaty; difficult to be the one manning the computer during a conference call in font of others” (No.26, Female, 32 years, palmar-plantar)

**Psychological Well-being:**

Negative emotions:

“...little things... when you are wearing a ring on your finger and people want to have a look so they grab your hand and you feel all embarrassed because they are sweaty; having your nails done, they are constantly working with your hands […] once again you feel embarrassed because you are sweating”. (No.70, Female, 21 years, palmar-plantar).

Peoples judgement:

“I can’t raise my hand all the way without showing my huge puddle of sweat...I can’t tell you how many times I heard "your back’s wet" from the person behind me. Just pure embarrassment” (*field notes: this was a student describing their experience of being in a lecture room*) (No. 16, Female, 17 years, axillar plus other sites)

“if you are giving something to someone and you have sweat marks all over it... its nasty...and it’s not something that you can control...” (No. 17, Female, 55 years, palmar-plantar)

Self-image:

“I have heard “everybody sweats while working out” way too many times, its way different when you are embarrassed because you are the only one looking like you took a shower, and everyone just has a tiny bit of sweat. I can’t raise my hand all the way without showing my huge puddle of sweat...I can’t tell you how many times I heard "your backs wet" from the person behind me. Just pure embarrassment”. (No.31, Female, 19 years, generalised).

“If you are giving something to someone and you’ve sweat marks all over it... its nasty...and it’s not something that you can control...” (No.20, Female, 58 years, palmar-plantar)

Restricted life and loss of control:

Everything you do in life you have to think the sweating will become a problem. Sports, activities such as walking, cycling, even driving, social events such as eating out, this can be a complete nightmare if it is a hot place where you go (No.1, Female, 24 years, palmar-plantar).

**“**when you sweat as severely as I do, often the only adaptation is to do nothing at all. Sitting still or laying down with a fan on is sometimes all I can do because my body is so hot that it’s in panic mode most of the time” (No.33, Female, 45 years, generalised).

**Social Life**

Being in social situations:

“Hyperhidrosis has also had an impact on social decisions in my life in the past. There have been times where I’ve been very nervous about going to a party or work gathering because of the fear of hyperhidrosis” (No.65, Female, 26 years, Axillar).

“Being at work, or travelling to and from work is mortifying. Sitting or standing in sodden clothes for 8 hours and travelling on public transport is horrendous. Have you ever dripped sweat on someone? Or watched someone recoil at the sight of rivers of sweat running down your face, hair and clothes? Or stating the obvious that am sweaty?” (No.33, Female, 45 years, generalised)

**Dealing with hyperhidrosis**

Personal hygiene and special chores:

“it makes you feel very unclean sometimes...you are constantly bathing...3 ...4...5 times a day. It takes over your life literary…” (No.13, Male, 45 years, generalised)

“Longer days in school was no fun. Being sweaty for long hours makes it harder to hide and it feels very disgusting /awkward being sweaty. And the fear that the sweat will start to smell. I tried to avoid these long days at school as much as possible or at least have two shirts with me to school, so I could change” (No.47, Male, 24 years, generalised).

Time and financial burden:

“… When I do take a shower I have to wait... like for an hour to completely dry ...or relax...not to do anything... It’s not nice at all” (No.3, Female, 18 years, Axillar plus other sites).

“I try not to be short of time to get to any appointment as rushing will cause a problem – as soon as you arrive and stop rushing, that’s when the head will leak (No.2, Female, 52 years, Cranial-facial).

**Unmet needs**

Clinical management of hyperhidrosis:

“my GP [general practitioner] didn’t diagnose that I had hyperhidrosis or identify that it was a condition...even though I took a magazine article along...he sort of acted like it was rubbish...and just prescribed me a whole lot of ahhh... told me to put a lot more of anti-perspirants on...told me to get a stronger antiperspirants...it was quite humiliating...” (No.9, Male, 38 years, palmar-plantar).

Lack of effective treatments:

Patients described how systemic treatments left them with a dry-mouth; iontopheresis caused skin thinning, irritation and a bitter taste in the mouth and the high risk for compensatory sweating and interference with emotional functioning associated with surgery.

Information needs:

“The lack of knowledge about hyperhidrosis among the medical community is also frustrating... I feel that it is difficult to find a provider that is knowledgeable about this condition because it is not a "sexy" diagnosis, there is so little funding that goes into research for hyperhidrosis. I am very thankful for research such as this that allows for any insight into living with hyperhidrosis” (No.65, Female, 26 years, Axillar)

**Daily life**

Everyday activities:

My life revolves around how sweaty I am that day. If I’m really sweaty, I stay inside and to myself. If I’m not too sweaty, I will more than likely go do something. It affects how I pay for things. I don't use cash anymore unless I know I have the exact change, because I don't like getting coins back since the other person usually touches your hand when they give you change back (No. 51, Female, 25 years, palmar-plantar).

“… at home [the sweating] mostly interferes with activities that require me to hold things like reading a newspaper, I can’t do that without putting the paper down lest I soak it. Also, needlework or crocheting can get difficult because my grip on the needles isn’t so good and I make the wool wet. I’ve ruined at least one mobile phone with water damage” (No.68, Female, 22 years, generalised).

**Dealing with hyperhidrosis**

Special chores and hygiene:

I still to this day try to keep my arms pinned to my sides in case the sweating shows through. We become masters of disguise... I used to hide under layers of clothing even in the hot summers. Just to hide the sweat (No.66, Female, 40 years, generalised)

Time and financial burden:

“it [hyperhidrosis] is burdensome. continuous buying of Driclor [20% aluminium chloride hexahydrate] and antiperspirants, antibacterial soaps, extra sandals and socks, etc. is indeed expensive and not a normal choice… it literally becomes part of your budget, we simply have no choice. (No.61, Female, 41 years, palmar-plantar).

**Professional or School Life**

Work and school tasks:

“Jobs are difficult as you can imagine handing someone’s change back wet, as they look at the beads of sweat on your hands reflecting under the light or when you are writing and the paper sticks to your hand and smudges the ink” (No.30, Male, 31 years, Axillar).

Being nervous about public speaking only makes the whole thing worse although I think I’m mostly nervous about the sweating which makes me sweat more of course. I hate the field work that’s part of my course because then I’m under flaming sun sweating more than ever, walking around with sweaty feet and having to talk to strangers plus take notes” (*field notes: this was a student*) (No.68, Female, 22 years, generalised).

…school is [was] very difficult because of it [hyperhidrosis] not only for the writing…you were always pulling your sleeve up underneath your hand or putting paper underneath your hand… so that the sweat in your hands doesn’t mark your books…but also because other teenagers are quick to pick on anything different or anything that looks different “(No.9, Male, 38 years, palmar-plantar).

“as a musician when I play the piano that was pretty hard…when am doing performance or even practicing its quite difficult…it’s something you have to live with (No.18., Male, 25 years, Palmar-plantar)

“pharmacist holding out change or giving prescriptions to patients” (No.21, female, 44 years, palmar-plantar).

“unable to meet with clients and panic if called into a meeting even if with only workmates” (No. 46, male, 37 years, Axillar).

“in college I am a performing arts student, so I have to move around a lot. I have to perform in front of an audience, which makes me nervous as it is, but sweat adds to my nerves and restricts may acting capabilities (No. 16, Female, 17 years, Axillar plus other sites).

“being in an office environment; especially open working spaces is difficult”. (No. 46, male, 37 years, Axillar).

“I am taking a Spanish course now which is much worse, often the teacher can ask unprepared questions which makes me a bit stressed making me sweat. Therefore, I don’t go to most of these lectures anymore (No.47, male, 24 years, generalised).

Career:

“if I am to be absolutely honest...when I was at school I would have applied to do medicine...but because I knew I would have to examine people with my hands which I could never do I opted to do pharmacy because I knew I would be able to do that because I was pretty academic” (No. 21, Female, 44 years, palmar-plantar).

“Participant [xx] really hit the nail on the head about taking less chances in life because of hyperhidrosis and the fact that it will even affect job prospects. Let’s face it, networking and impression that you make on people determine career progress and looking like a human fountain, constantly fumbling with hankies and soaking through clothes, doesn’t make the best impression” (No.68, Female, 22 years, generalised)”.
